# Supplementary material for: Hybrid Models and Biological Model Reduction with PyDSTool
Source: PLoS Comput Biol. 2012 Aug 9;8(8):e1002628. doi: 10.1371/journal.pcbi.1002628 (PMC3415397; doi:10.1371/journal.pcbi.1002628)
Supplement: Text S4 — Complete source code for the PyDSTool package (version 0.88.120504). Includes API documentation and help files linking to web pages. This file is identical to the current public release on Sourceforge.net. (ZIP) [file pcbi.1002628.s004.zip › PyDSTool/html/PyDSTool.Symbolic-module.html]

xml version="1.0" encoding="ascii"?


PyDSTool.Symbolic


| Home | Trees | Indices | Help | PyDSTool |
| --- | --- | --- | --- | --- |

Package PyDSTool ::
Module Symbolic

[hide private]

[frames] | no frames]

# Module Symbolic

source code

```
Symbolic expression support, and associated utilities.

Robert Clewley, October 2005.

   Includes code for symbolic differentiation by Pearu Peterson
   and Ryan Gutenkunst.


 Symbolic Differentiation code:
    Copyright 1999 Pearu Peterson, <pearu@ioc.ee>
    March 11-12, 1999
    Pearu Peterson

    Modified by Ryan Gutenkunst, <rng7@cornell.edu>
    April 28th, 2005
    General clean-up of code.
    Also changed saving mechanism to explicitly save the results,
    rather than doing it each time a derivative is taken.

    Modified by Robert Clewley, <rhc28@cornell.edu>
    September 15th, 2005
    As this branch of Peterson's development of symbolic features in Python
      seems to be stopped, I have increased the version number to 0.3 from 0.2.
    Added division by zero check in function dodiv().
    Added decimal point to integer constants in division to avoid Python
      integer division rules being applied to the expressions. This involves
      an added optional argument to trysimple(), and a new function
      ensuredecimalconst(). This option is conrolled using the global DO_DEC
      switch.
    Added functionality in dofun() for using "pow(x,y)" (with two arguments only)
      in input expressions.
    Also, added DO_POW option to make all output involving powers to use this
      "pow(x,y)" syntax, rather than "x**y". (Set the global DO_POW switch).
    dopower(), domul(), etc. also accept decimals 1.0 and 0.0 to perform
      simplifications if the DO_DEC switch is set.
    Adapted Diff function to accept PyDSTool Var and QuantSpec types, and
      always return those types, and also to accept an array (acts like Jacobian).
      Code is entirely backwards compatible if PyDSTool is not used (except
      Jacobians won't work).
    Renamed original, pure-string Diff function to DiffStr.
    test3() augmented with test for "pow(x,y)" syntax.
    Added test4() for Diff as Jacobian and using Var and QuantSpec objects,
      if available.
    Note that with DO_DEC option off you get "incorrect" results for the derivative
      of f[1] in test4().

    25 February 2006
    Moved package into Symbolic.py
    Added scopes to eval statements to prevent argument names clashing with
      names appearing in the eval'd string.
    Made title case symbolic versions of math functions and constants
      from ModelSpec compatible with Diff.
    Improved support for pow(x,y) syntax under differentiation.
    Added support for symbolic vectors.
```


|  |  |
| --- | --- |
| Classes | [hide private] |

\_mathobj

QuantSpec  
Specification for a symbolic numerical quantity.

Quantity  
Abstract class for symbolic numerical quantities.

Par

Var

Input

Fun


|  |  |
| --- | --- |
| Functions | [hide private] |

|  |  |
| --- | --- |
| resolveSpecTypeCombos(t1, t2)  Resolve Quantity and QuantSpec types when combined. | source code |

|  |  |
| --- | --- |
| getdim(s)  If s is a representation of a vector, return the dimension. | source code |

|  |  |
| --- | --- |
| strIfSeq(x)  Convert sequence of QuantSpecs or strings to a string representation of that sequence, otherwise return the argument untouched. | source code |

|  |  |
| --- | --- |
| ensureQlist(thelist) | source code |

|  |  |
| --- | --- |
| ensureStrArgDict(d, renderForCode=True)  Optional renderForCode argument converts any uppercase built-in symbolic function names to lowercase numeric function names (default True) | source code |

|  |  |
| --- | --- |
| \_eval(q, math\_globals, local\_free, eval\_at\_runtime) | source code |

|  |  |
| --- | --- |
| \_propagate\_str\_eval(s) | source code |

|  |  |
| --- | --- |
| \_join\_sublist(qspec, math\_globals, local\_free, eval\_at\_runtime) | source code |

|  |  |
| --- | --- |
| expr2fun(qexpr, ensure\_args=None, \*\*values)  qexpr is a string, Quantity, or QuantSpec object. | source code |

|  |  |
| --- | --- |
| subs(qexpr, \*bindings)  Substitute pars or other name bindings (as a dictionary) into a Quantity, QuantSpec, or string containing a symbolic expression. | source code |

|  |  |
| --- | --- |
| \_parse\_func\_deriv(fname)  Internal utility to test whether a function name is generated from a symbolic differentiation call. | source code |

|  |  |
| --- | --- |
| \_generate\_subderivatives(symbols, fnspecs)  Internal utility for prepJacobian | source code |

|  |  |
| --- | --- |
| prepJacobian(varspecs, coords, fnspecs=None, max\_iter\_depth=20)  Returns a symbolic Jacobian and updated function specs to support its definition from variable specifications. | source code |

|  |  |
| --- | --- |
| checkbraces(s)  Internal utility to verify that braces match. | source code |

|  |  |
| --- | --- |
| whoQ(objdict=None, deepSearch=False, frameback=3) | source code |

|  |  |
| --- | --- |
| processMultiDef(s, specstr, specType) | source code |

|  |  |
| --- | --- |
| isMultiDef(s)  Determine whether string is a candidate multiple Quantity definition. | source code |

|  |  |
| --- | --- |
| findMultiRefs(s) | source code |

|  |  |
| --- | --- |
| isMultiRef(s)  Simple litmus test for candidate multiple quantity reference (or definition). | source code |

|  |  |
| --- | --- |
| processMultiRef(s, rootname, ixname, ok\_range) | source code |

|  |  |
| --- | --- |
| isMultiDefClash(obj, multiDefRefs, parentname=None) | source code |

|  |  |
| --- | --- |
| evalMultiRefToken(mref, ixname, val)  Helper function for evaluating multi-reference tokens for given index values. | source code |

|  |  |
| --- | --- |
| getlftrtD(t, a) | source code |

|  |  |
| --- | --- |
| dofun(l, r) | source code |

|  |  |
| --- | --- |
| Diff(t, a)  Diff expects strings, Quantity, or QuantSpec types, a mixture of these, or a list of strings in second argument. | source code |

|  |  |
| --- | --- |
| DiffStr(t, a=`'``x``'`)  Diff for strings only. | source code |


|  |  |
| --- | --- |
| Variables | [hide private] |

builtin\_auxnames = `['globalindepvar', 'initcond', 'heav', 'if'...`

re

replaceSepStr

num\_chars = `['0', '1', '2', '3', '4', '5', '6', '7', '8', '9']`

builtinFnSigInfo = `{'for': 4, 'getbound': 2, 'getindex': 1, 'g...`

token

specialfns

alphabet\_chars\_RE

collect\_numbers

toCircumflexSyntax

syms = `{0: 'ENDMARKER', 1: 'NAME', 2: 'NUMBER', 3: 'STRING', 4...`

protected\_allnames = `['acos', 'asin', 'atan', 'atan2', 'ceil',...`

ZEROS = `['0', '0.0', '0.', '(0)', '(0.0)', '(0.)']`

reserved\_keywords

isVectorClause

protected\_mathnames = `['acos', 'asin', 'atan', 'atan2', 'ceil'...`

parser

temp\_macro\_names

replaceSepQSpec

protected\_macronames = `['for', 'if', 'max', 'min', 'sum']`

protected\_auxnamesDB = `ModelSpec internal helper class: auxfnD...`

DO\_DEC

protected\_specialfns = `['special_airy', 'special_airye', 'spec...`

ensureints

convert\_power\_reserved\_keywords

name\_chars\_RE = `re.compile(r'\w')`

protected\_randomnames = `['BPF', 'LOG4', 'NV_MAGICCONST', 'RECI...`

temp\_macro\_names\_inv

protected\_scipynames = `['sign', 'mod']`

toDoubleStarSyntax

symbol

TENS

POW\_STR

NAMESEP = `'.'`

ONES = `['1', '1.0', '1.', '(1)', '(1.0)', '(1.)']`

DO\_POW

all

predicate\_op

less

shape

complexfloating

DomainType

putmask

swapaxes

newton\_meth

float32

take

float

greater

float96

complex\_

ones

API = `API_class()`

targetLangs = `['c', 'python', 'matlab']`

concatenate

API\_class

argmax

sign

Macheps

mat

asarray

Continuous = `Continuous Domain`

null\_predicate = `null_predicate_class(None)`

complex64

transpose

atleast\_1d

Discrete = `Discrete Domain`

float\_

less\_equal

complex128

zeros

int\_

complex192

int0

int8

argmin

double

norm

finfo

integer

spy

clip

int

logical\_or

floating

argsort

any

null\_predicate\_class

rout

redirc

complex

LargestInt32 = `2147483647`

int64

int16

npy

minpack

ndarray

searchsorted

fit\_function

time

rerr

Inf = `inf`

NaN = `nan`

isfinite = `<ufunc 'isfinite'>`

mod = `<ufunc 'remainder'>`

arccos = `<ufunc 'arccos'>`

arcsin = `<ufunc 'arcsin'>`

arctan = `<ufunc 'arctan'>`

arctan2 = `<ufunc 'arctan2'>`

arccosh = `<ufunc 'arccosh'>`

arcsinh = `<ufunc 'arcsinh'>`

arctanh = `<ufunc 'arctanh'>`

ceil = `<ufunc 'ceil'>`

cos = `<ufunc 'cos'>`

cosh = `<ufunc 'cosh'>`

exp = `<ufunc 'exp'>`

fabs = `<ufunc 'fabs'>`

floor = `<ufunc 'floor'>`

fmod = `<ufunc 'fmod'>`

frexp = `<ufunc 'frexp'>`

hypot = `<ufunc 'hypot'>`

ldexp = `<ufunc 'ldexp'>`

log = `<ufunc 'log'>`

log10 = `<ufunc 'log10'>`

modf = `<ufunc 'modf'>`

power = `<ufunc 'power'>`

sin = `<ufunc 'sin'>`

sinh = `<ufunc 'sinh'>`

sqrt = `<ufunc 'sqrt'>`

tan = `<ufunc 'tan'>`

tanh = `<ufunc 'tanh'>`

pi = `3.14159265359`

e = `2.71828182846`

asin = `<ufunc 'arcsin'>`

acos = `<ufunc 'arccos'>`

atan = `<ufunc 'arctan'>`

atan2 = `<ufunc 'arctan2'>`

acosh = `<ufunc 'arccosh'>`

asinh = `<ufunc 'arcsinh'>`

atanh = `<ufunc 'arctanh'>`

pow = `<ufunc 'power'>`

math\_dir = `['__doc__', '__file__', '__name__', 'acos', 'asin',...`

math\_globals = `{'Abs': Abs (ModelSpec wrapper), 'Acos': Acos (...`

allmathnames = `['acos', 'asin', 'atan', 'atan2', 'ceil', 'cos'...`

allmathnames\_symbolic = `['Acos', 'Asin', 'Atan', 'Atan2', 'Cei...`

mathNameMap = `{'Abs': 'abs', 'Acos': 'acos', 'Asin': 'asin', '...`

inverseMathNameMap = `{'Random': 'Random', 'SystemRandom': 'Sys...`

specTypes = `('RHSfuncSpec', 'ImpFuncSpec', 'ExpFuncSpec')`

\_functions = `['isMultiDef', 'isMultiRef', 'isMultiDefClash', '...`

\_classes = `['QuantSpec', 'Quantity', 'Var', 'Par', 'Input', 'F...`

\_constants = `['specTypes', 'allmathnames_symbolic', 'allmathna...`

feval\_map\_const = `{'e': '2.7182818284590451', 'pi': '3.1415926...`

feval\_map\_symb = `{'Abs': 'abs', 'Acos': 'acos', 'Asin': 'asin'...`

mathlookup = `{'acos': 'math.', 'asin': 'math.', 'atan': 'math....`

randomlookup = `{'BPF': 'random.', 'LOG4': 'random.', 'NV_MAGIC...`

builtinlookup = `{'abs': '', 'max': '', 'min': '', 'pow': '', '...`

modlookup = `{'BPF': 'random.', 'LOG4': 'random.', 'NV_MAGICCON...`

funcnames = `['acos', 'asin', 'atan', 'atan2', 'ceil', 'cos', '...`

\_localfuncnames = `['for', 'if', 'max', 'min', 'sum', 'globalin...`

\_avoid\_math\_symbols = `['e', 'pi', 'Acos', 'Asin', 'Atan', 'Ata...`

local\_fndef = `{'for_': Fun for_ (ExpFuncSpec), 'getbound_': Fu...`

qtypes = `(<class 'PyDSTool.Symbolic.Quantity'>, <class 'PyDSTo...`

Abs = `Abs (ModelSpec wrapper)`

Acos = `Acos (ModelSpec wrapper)`

Asin = `Asin (ModelSpec wrapper)`

Atan = `Atan (ModelSpec wrapper)`

Atan2 = `Atan2 (ModelSpec wrapper)`

Betavariate = `Betavariate (ModelSpec wrapper)`

Ceil = `Ceil (ModelSpec wrapper)`

Choice = `Choice (ModelSpec wrapper)`

Cos = `Cos (ModelSpec wrapper)`

Cosh = `Cosh (ModelSpec wrapper)`

Degrees = `Degrees (ModelSpec wrapper)`

E = `QuantSpec e (ExpFuncSpec)`

Exp = `Exp (ModelSpec wrapper)`

Expovariate = `Expovariate (ModelSpec wrapper)`

Fabs = `Fabs (ModelSpec wrapper)`

Floor = `Floor (ModelSpec wrapper)`

Fmod = `Fmod (ModelSpec wrapper)`

Frexp = `Frexp (ModelSpec wrapper)`

Gammavariate = `Gammavariate (ModelSpec wrapper)`

Gauss = `Gauss (ModelSpec wrapper)`

Getrandbits = `Getrandbits (ModelSpec wrapper)`

Getstate = `Getstate (ModelSpec wrapper)`

Hypot = `Hypot (ModelSpec wrapper)`

Jumpahead = `Jumpahead (ModelSpec wrapper)`

Ldexp = `Ldexp (ModelSpec wrapper)`

Log = `Log (ModelSpec wrapper)`

Log10 = `Log10 (ModelSpec wrapper)`

Lognormvariate = `Lognormvariate (ModelSpec wrapper)`

Max = `Max (ModelSpec wrapper)`

Min = `Min (ModelSpec wrapper)`

Modf = `Modf (ModelSpec wrapper)`

Normalvariate = `Normalvariate (ModelSpec wrapper)`

Paretovariate = `Paretovariate (ModelSpec wrapper)`

Pi = `QuantSpec pi (ExpFuncSpec)`

Pow = `Pow (ModelSpec wrapper)`

Radians = `Radians (ModelSpec wrapper)`

Randint = `Randint (ModelSpec wrapper)`

Random = `Random (ModelSpec wrapper)`

Randrange = `Randrange (ModelSpec wrapper)`

Sample = `Sample (ModelSpec wrapper)`

Seed = `Seed (ModelSpec wrapper)`

Setstate = `Setstate (ModelSpec wrapper)`

Shuffle = `Shuffle (ModelSpec wrapper)`

Sin = `Sin (ModelSpec wrapper)`

Sinh = `Sinh (ModelSpec wrapper)`

Sqrt = `Sqrt (ModelSpec wrapper)`

Sum = `Sum (ModelSpec wrapper)`

Systemrandom = `Systemrandom (ModelSpec wrapper)`

Tan = `Tan (ModelSpec wrapper)`

Tanh = `Tanh (ModelSpec wrapper)`

Uniform = `Uniform (ModelSpec wrapper)`

Vonmisesvariate = `Vonmisesvariate (ModelSpec wrapper)`

Weibullvariate = `Weibullvariate (ModelSpec wrapper)`

Wichmannhill = `Wichmannhill (ModelSpec wrapper)`

\_1DimplicitSolveMethods = `['newton', 'bisect', 'steffe']`

\_all\_complex = `(<type 'complex'>, <type 'numpy.complexfloating...`

\_all\_float = `(<type 'float'>, <type 'numpy.floating'>, <type '...`

\_all\_int = `(<type 'int'>, <type 'numpy.integer'>, <type 'numpy...`

\_all\_numpy\_complex = `(<type 'numpy.complex128'>, <type 'numpy....`

\_all\_numpy\_float = `(<type 'numpy.float64'>, <type 'numpy.float...`

\_all\_numpy\_int = `(<type 'numpy.int32'>, <type 'numpy.int32'>, ...`

\_complex\_types = `(<type 'complex'>, <type 'numpy.complexfloati...`

\_float\_types = `(<type 'float'>, <type 'numpy.floating'>)`

\_implicitSolveMethods = `['newton', 'bisect', 'steffe', 'fsolve']`

\_indentstr = `' '`

\_int\_types = `(<type 'int'>, <type 'numpy.integer'>)`

\_num\_equivtype = `{<type 'float'>: <type 'numpy.float64'>, <typ...`

\_num\_maxmin = `{<type 'numpy.int32'>: [-2147483648, 2147483647]...`

\_num\_name2equivtypes = `{'float': (<type 'float'>, <type 'numpy...`

\_num\_name2type = `{'float': <type 'numpy.float64'>, 'int': <typ...`

\_num\_type2name = `{<type 'float'>: 'float', <type 'int'>: 'int'...`

\_num\_types = `(<type 'float'>, <type 'int'>, <type 'numpy.float...`

\_pytypefromtype = `{<type 'numpy.int32'>: <type 'int'>, <type '...`

\_real\_types = `(<type 'int'>, <type 'numpy.integer'>, <type 'fl...`

\_seq\_types = `(<type 'list'>, <type 'tuple'>, <type 'numpy.ndar...`

a = `'sum'`

argstr = `'\'arg0\',\'arg1\',\'arg2\','`

c = `'pi'`

f = `'sum'`

fname = `'if'`

funcstr = `'Fun(\'\', [\'arg0\',\'arg1\',\'arg2\',], \'if_\')'`

i = `2`

m = `'tanh'`

n = `'sum'`

symb = `'Sum'`


|  |  |
| --- | --- |
| Function Details | [hide private] |

|  |  |
| --- | --- |
| resolveSpecTypeCombos(t1, t2) | source code |

Resolve Quantity and QuantSpec types when combined. QuantSpec
<op> QuantSpec -> specType rules: same types -> that type
ExpFuncSpec o RHSfuncSpec -> RHSfuncSpec ImpFuncSpec o RHSfuncSpec
-> RHSfuncSpec (should this be an error?) ImpFuncSpec o ExpFuncSpec
-> ImpFuncSpec

|  |  |
| --- | --- |
| expr2fun(qexpr, ensure\_args=None, \*\*values) | source code |

qexpr is a string, Quantity, or QuantSpec object. values is a
dictionary binding free names to numerical values or other objects
defined at runtime that are in local scope.

If values contains pairs (fnsig, fndef) from a FuncSpec function
definition then the function will be incorporated into the result. The
function definition can contain math names that are still in
non-code-rendered (lowercase) form (i.e. "Exp" rather than
"exp").

If any names use the hierarchical naming convention (using the '.'
separator) then those names will be converted to use the '\_' separator
instead. For end-use convenience, the necessary name map for arguments is
stored in the '\_namemap' attribute, which will otherwise be the ID map.
Calling with keyed objects that use hierarchical names (especially Point
objects) can then be done using the return object's alt\_call method,
which takes any keyed\_object that will be filtered for the function
arguments and name-mapped appropriately before evaluation.

You can force the function to accept the named arguments in the
ensure\_args list of strings, e.g. when creating a Jacobian that is
expected to always take 't' as an argument even if the system is
autonomous.

The arguments to the returned callable function object are given by
its attribute '\_args', and its definition string by '\_call\_spec'.

|  |  |
| --- | --- |
| prepJacobian(varspecs, coords, fnspecs=None, max\_iter\_depth=20) | source code |

Returns a symbolic Jacobian and updated function specs to support its
definition from variable specifications. Only makes the Jacobian with
respect to the named coordinates, which will be sorted into alphabetical
order.

|  |  |
| --- | --- |
| isMultiRef(s) | source code |

Simple litmus test for candidate multiple quantity reference (or
definition).

Requires further testing using one of the other associated multiple
quantity reference/definition tests.

|  |  |
| --- | --- |
| Diff(t, a) | source code |

Diff expects strings, Quantity, or QuantSpec types, a mixture of
these, or a list of strings in second argument.

The return type is a QuantSpec.

|  |  |
| --- | --- |
| DiffStr(t, a=`'``x``'`) | source code |

Diff for strings only.

This is Pearu's original Diff function renamed to DiffStr.

  


|  |  |
| --- | --- |
| Variables Details | [hide private] |

### builtin\_auxnames

Value:
:   ```
    ['globalindepvar', 'initcond', 'heav', 'if', 'getindex', 'getbound']
    ```

### builtinFnSigInfo

Value:
:   ```
    {'for': 4,
     'getbound': 2,
     'getindex': 1,
     'globalindepvar': 1,
     'heav': 1,
     'if': 3,
     'initcond': 1,
     'max': 1,
    ...
    ```

### syms

Value:
:   ```
    {0: 'ENDMARKER',
     1: 'NAME',
     2: 'NUMBER',
     3: 'STRING',
     4: 'NEWLINE',
     5: 'INDENT',
     6: 'DEDENT',
     7: 'LPAR',
    ...
    ```

### protected\_allnames

Value:
:   ```
    ['acos',
     'asin',
     'atan',
     'atan2',
     'ceil',
     'cos',
     'cosh',
     'degrees',
    ...
    ```

### protected\_mathnames

Value:
:   ```
    ['acos',
     'asin',
     'atan',
     'atan2',
     'ceil',
     'cos',
     'cosh',
     'degrees',
    ...
    ```

### protected\_auxnamesDB

Value:
:   ```
    ModelSpec internal helper class: auxfnDBclass object
    ```

### protected\_specialfns

Value:
:   ```
    ['special_airy',
     'special_airye',
     'special_ai_zeros',
     'special_bi_zeros',
     'special_ellipj',
     'special_ellipk',
     'special_ellipkinc',
     'special_ellipe',
    ...
    ```

### protected\_randomnames

Value:
:   ```
    ['BPF',
     'LOG4',
     'NV_MAGICCONST',
     'RECIP_BPF',
     'Random',
     'SG_MAGICCONST',
     'SystemRandom',
     'TWOPI',
    ...
    ```

### math\_dir

Value:
:   ```
    ['__doc__',
     '__file__',
     '__name__',
     'acos',
     'asin',
     'atan',
     'atan2',
     'ceil',
    ...
    ```

### math\_globals

Value:
:   ```
    {'Abs': Abs (ModelSpec wrapper),
     'Acos': Acos (ModelSpec wrapper),
     'Asin': Asin (ModelSpec wrapper),
     'Atan': Atan (ModelSpec wrapper),
     'Atan2': Atan2 (ModelSpec wrapper),
     'Betavariate': Betavariate (ModelSpec wrapper),
     'Ceil': Ceil (ModelSpec wrapper),
     'Choice': Choice (ModelSpec wrapper),
    ...
    ```

### allmathnames

Value:
:   ```
    ['acos',
     'asin',
     'atan',
     'atan2',
     'ceil',
     'cos',
     'cosh',
     'degrees',
    ...
    ```

### allmathnames\_symbolic

Value:
:   ```
    ['Acos',
     'Asin',
     'Atan',
     'Atan2',
     'Ceil',
     'Cos',
     'Cosh',
     'Degrees',
    ...
    ```

### mathNameMap

Value:
:   ```
    {'Abs': 'abs',
     'Acos': 'acos',
     'Asin': 'asin',
     'Atan': 'atan',
     'Atan2': 'atan2',
     'Betavariate': 'betavariate',
     'Ceil': 'ceil',
     'Choice': 'choice',
    ...
    ```

### inverseMathNameMap

Value:
:   ```
    {'Random': 'Random',
     'SystemRandom': 'Systemrandom',
     'WichmannHill': 'Wichmannhill',
     'abs': 'Abs',
     'acos': 'Acos',
     'asin': 'Asin',
     'atan': 'Atan',
     'atan2': 'Atan2',
    ...
    ```

### \_functions

Value:
:   ```
    ['isMultiDef',
     'isMultiRef',
     'isMultiDefClash',
     'checkbraces',
     'findMultiRefs',
     'processMultiRef',
     'evalMultiRefToken',
     'expr2fun',
    ...
    ```

### \_classes

Value:
:   ```
    ['QuantSpec', 'Quantity', 'Var', 'Par', 'Input', 'Fun']
    ```

### \_constants

Value:
:   ```
    ['specTypes',
     'allmathnames_symbolic',
     'allmathnames',
     'Acos',
     'Asin',
     'Atan',
     'Atan2',
     'Ceil',
    ...
    ```

### feval\_map\_const

Value:
:   ```
    {'e': '2.7182818284590451', 'pi': '3.1415926535897931'}
    ```

### feval\_map\_symb

Value:
:   ```
    {'Abs': 'abs',
     'Acos': 'acos',
     'Asin': 'asin',
     'Atan': 'atan',
     'Atan2': 'atan2',
     'Betavariate': 'betavariate',
     'Ceil': 'ceil',
     'Choice': 'choice',
    ...
    ```

### mathlookup

Value:
:   ```
    {'acos': 'math.',
     'asin': 'math.',
     'atan': 'math.',
     'atan2': 'math.',
     'ceil': 'math.',
     'cos': 'math.',
     'cosh': 'math.',
     'degrees': 'math.',
    ...
    ```

### randomlookup

Value:
:   ```
    {'BPF': 'random.',
     'LOG4': 'random.',
     'NV_MAGICCONST': 'random.',
     'RECIP_BPF': 'random.',
     'Random': 'random.',
     'SG_MAGICCONST': 'random.',
     'SystemRandom': 'random.',
     'TWOPI': 'random.',
    ...
    ```

### builtinlookup

Value:
:   ```
    {'abs': '', 'max': '', 'min': '', 'pow': '', 'sum': ''}
    ```

### modlookup

Value:
:   ```
    {'BPF': 'random.',
     'LOG4': 'random.',
     'NV_MAGICCONST': 'random.',
     'RECIP_BPF': 'random.',
     'Random': 'random.',
     'SG_MAGICCONST': 'random.',
     'SystemRandom': 'random.',
     'TWOPI': 'random.',
    ...
    ```

### funcnames

Value:
:   ```
    ['acos',
     'asin',
     'atan',
     'atan2',
     'ceil',
     'cos',
     'cosh',
     'degrees',
    ...
    ```

### \_localfuncnames

Value:
:   ```
    ['for',
     'if',
     'max',
     'min',
     'sum',
     'globalindepvar',
     'initcond',
     'heav',
    ...
    ```

### \_avoid\_math\_symbols

Value:
:   ```
    ['e',
     'pi',
     'Acos',
     'Asin',
     'Atan',
     'Atan2',
     'Ceil',
     'Cos',
    ...
    ```

### local\_fndef

Value:
:   ```
    {'for_': Fun for_ (ExpFuncSpec),
     'getbound_': Fun getbound_ (ExpFuncSpec),
     'getindex_': Fun getindex_ (ExpFuncSpec),
     'globalindepvar_': Fun globalindepvar_ (ExpFuncSpec),
     'heav_': Fun heav_ (ExpFuncSpec),
     'if_': Fun if_ (ExpFuncSpec),
     'initcond_': Fun initcond_ (ExpFuncSpec),
     'max_': Fun max_ (ExpFuncSpec),
    ...
    ```

### qtypes

Value:
:   ```
    (<class 'PyDSTool.Symbolic.Quantity'>,
     <class 'PyDSTool.Symbolic.QuantSpec'>)
    ```

### \_all\_complex

Value:
:   ```
    (<type 'complex'>,
     <type 'numpy.complexfloating'>,
     <type 'numpy.complex128'>,
     <type 'numpy.complex64'>,
     <type 'numpy.complex128'>)
    ```

### \_all\_float

Value:
:   ```
    (<type 'float'>,
     <type 'numpy.floating'>,
     <type 'numpy.float64'>,
     <type 'numpy.float32'>,
     <type 'numpy.float64'>)
    ```

### \_all\_int

Value:
:   ```
    (<type 'int'>,
     <type 'numpy.integer'>,
     <type 'numpy.int32'>,
     <type 'numpy.int32'>,
     <type 'numpy.int8'>,
     <type 'numpy.int16'>,
     <type 'numpy.int32'>,
     <type 'numpy.int64'>)
    ```

### \_all\_numpy\_complex

Value:
:   ```
    (<type 'numpy.complex128'>,
     <type 'numpy.complex64'>,
     <type 'numpy.complex128'>)
    ```

### \_all\_numpy\_float

Value:
:   ```
    (<type 'numpy.float64'>,
     <type 'numpy.float32'>,
     <type 'numpy.float64'>)
    ```

### \_all\_numpy\_int

Value:
:   ```
    (<type 'numpy.int32'>,
     <type 'numpy.int32'>,
     <type 'numpy.int8'>,
     <type 'numpy.int16'>,
     <type 'numpy.int32'>,
     <type 'numpy.int64'>)
    ```

### \_complex\_types

Value:
:   ```
    (<type 'complex'>, <type 'numpy.complexfloating'>)
    ```

### \_num\_equivtype

Value:
:   ```
    {<type 'float'>: <type 'numpy.float64'>,
     <type 'int'>: <type 'numpy.int32'>,
     <type 'numpy.integer'>: <type 'numpy.int32'>,
     <type 'numpy.floating'>: <type 'numpy.float64'>,
     <type 'numpy.int8'>: <type 'numpy.int32'>,
     <type 'numpy.int16'>: <type 'numpy.int32'>,
     <type 'numpy.int32'>: <type 'numpy.int32'>,
     <type 'numpy.int32'>: <type 'numpy.int32'>,
    ...
    ```

### \_num\_maxmin

Value:
:   ```
    {<type 'numpy.int32'>: [-2147483648, 2147483647],
     <type 'numpy.float64'>: [-inf, inf]}
    ```

### \_num\_name2equivtypes

Value:
:   ```
    {'float': (<type 'float'>,
               <type 'numpy.floating'>,
               <type 'numpy.float64'>,
               <type 'numpy.float32'>,
               <type 'numpy.float64'>),
     'int': (<type 'int'>,
             <type 'numpy.integer'>,
             <type 'numpy.int32'>,
    ...
    ```

### \_num\_name2type

Value:
:   ```
    {'float': <type 'numpy.float64'>, 'int': <type 'numpy.int32'>}
    ```

### \_num\_type2name

Value:
:   ```
    {<type 'float'>: 'float',
     <type 'int'>: 'int',
     <type 'numpy.integer'>: 'int',
     <type 'numpy.floating'>: 'float',
     <type 'numpy.int8'>: 'int',
     <type 'numpy.int16'>: 'int',
     <type 'numpy.int32'>: 'int',
     <type 'numpy.int32'>: 'int',
    ...
    ```

### \_num\_types

Value:
:   ```
    (<type 'float'>,
     <type 'int'>,
     <type 'numpy.floating'>,
     <type 'numpy.integer'>)
    ```

### \_pytypefromtype

Value:
:   ```
    {<type 'numpy.int32'>: <type 'int'>,
     <type 'numpy.float64'>: <type 'float'>}
    ```

### \_real\_types

Value:
:   ```
    (<type 'int'>,
     <type 'numpy.integer'>,
     <type 'float'>,
     <type 'numpy.floating'>)
    ```

### \_seq\_types

Value:
:   ```
    (<type 'list'>, <type 'tuple'>, <type 'numpy.ndarray'>)
    ```

  


| Home | Trees | Indices | Help | PyDSTool |
| --- | --- | --- | --- | --- |

|  |  |
| --- | --- |
| Generated by Epydoc 3.0.1 on Fri May 4 15:24:03 2012 | http://epydoc.sourceforge.net |
